# Transcription factors Sp1 and Sp4 regulate TRPV1 gene expression in rat sensory neurons

**DOI:** 10.1186/1744-8069-7-44

**Published:** 2011-06-06

**Authors:** Catherine Chu, Kathryn Zavala, Atefeh Fahimi, Jessica Lee, Qing Xue, Helge Eilers, Mark A Schumacher

**Affiliations:** 1 University of California, San Francisco Department of Anesthesia and Perioperative Care 513 Parnassus Ave. Rm. S436 (Box0427), University of California, San Francisco 94143-0427 USA

## Abstract

**Background:**

The capsaicin receptor, transient receptor potential vanilloid type -1 (TRPV1) directs complex roles in signal transduction including the detection of noxious stimuli arising from cellular injury and inflammation. Under pathophysiologic conditions, TRPV1 mRNA and receptor protein expression are elevated in dorsal root ganglion (DRG) neurons for weeks to months and is associated with hyperalgesia. Building on our previous isolation of a promoter system for the rat TRPV1 gene, we investigated the proximal TRPV1 P2-promoter by first identifying candidate Sp1-like transcription factors bound *in vivo *to the P2-promoter using chromatin immunoprecipitation (ChIP) assay. We then performed deletion analysis of GC-box binding sites, and quantified promoter activity under conditions of Sp1 / Sp4 over-expression versus inhibition/knockdown. mRNA encoding Sp1, Sp4 and TRPV1 were quantified by qRT-PCR under conditions of Sp1/Sp4 over-expression or siRNA mediated knockdown in cultured DRG neurons.

**Results:**

Using ChIP analysis of DRG tissue, we demonstrated that Sp1 and Sp4 are bound to the candidate GC-box site region within the endogenous TRPV1 P2-promoter. Deletion of GC-box "a" or "a + b" within the P2- promoter resulted in a complete loss of transcriptional activity indicating that GC-box "a" was the critical site for promoter activation. Co-transfection of Sp1 increased P2-promoter activity in cultured DRG neurons whereas mithramycin-a, an inhibitor of Sp1-like function, dose dependently blocked NGF and Sp1-dependent promoter activity in PC12 cells. Co-transfection of siRNA directed against Sp1 or Sp4 decreased promoter activity in DRG neurons and NGF treated PC12 cells. Finally, electroporation of Sp1 or Sp4 cDNA into cultures of DRG neurons directed an increase in Sp1/Sp4 mRNA and importantly an increase in TRPV1 mRNA. Conversely, combined si-RNA directed knockdown of Sp1/Sp4 resulted in a decrease in TRPV1 mRNA.

**Conclusion:**

Based on these studies, we now propose a model of TRPV1 expression that is dependent on Sp1-like transcription factors with Sp4 playing a predominant role in activating TRPV1 RNA transcription in DRG neurons. Given that increases of TRPV1 expression have been implicated in a wide range of pathophysiologic states including persistent painful conditions, blockade of Sp1-like transcription factors represents a novel direction in therapeutic strategies.

## Background

Identification of receptors/ion channels that respond to noxious stimuli has been at the forefront of a new understanding of peripheral pain transduction. A seminal finding was the isolation of TRPV1 (capsaicin receptor, VR1) [1] which functions as an integrator of multiple noxious stimuli [2-5] and is essential for the detection of inflammatory pain/hyperalgesia [6,7]. TRPV1 is not only selectively expressed in a subset of primary afferent nociceptors, but its expression is also dynamically regulated. Nociceptor expression of TRPV1 mRNA and receptor protein is lost over a period of days when target-tissue derived trophic factors such as Nerve Growth Factor (NGF) are reduced [8,9]. In contrast, conditions that increase trophic factors as a result of inflammation or tissue-nerve injury result in an increase in TRPV1 mRNA and/or receptor protein expression [10-13]. In part, these reports suggest that a transcription-dependent mechanism drives persistent TRPV1 mediated pain and hyperalgesia.

To advance our understanding of how TRPV1 transcription is enhanced under pathophysiologic conditions, we initially isolated and characterized a dual promoter system for the rat TRPV1 gene [14]. These studies revealed that the proximal P2-promoter directed cell-type specific activity that was positively regulated by the trophic factor NGF [14]. Building on these observations, we now investigate the role of regulatory sites within the P2-promoter and attempt to identify candidate transcription factors that control the activity of the P2-promoter and apparently regulate the transcription of TRPV1 RNA in sensory neurons. Based on transcription factor binding studies in dorsal root ganglion (DRG), luciferase-based transcriptional assays in cultured sensory neurons and NGF treated PC12 cells and quantitative measurements of mRNA encoding Sp1-like factors and TRPV1, we propose a model of TRPV1 gene expression that is dependent on the action of at least two members of the Sp1-like transcription factor family, Sp1 and Sp4, acting at a specific GC-box binding site.

## Methods

### Chromatin Immunoprecipitation assay (ChIP)

Identification of Sp1-like transcription factor binding to the TRPV1 promoter in native rat dorsal root ganglion (DRG) chromatin was obtained using ChIP-IT^® ^Enzymatic (Active Motif, Carlsbad, CA) with the following modifications: Whole rat DRG or enriched DRG neurons were harvested on ice then dounce homogenized ten times in an ethanol - dry ice bath followed by crosslinking (1% formaldehyde in PBS). Goat IgG antiserum directed against Sp1 (PEP 2), Sp3 (D-20) and Sp4 (V-20) (Santa Cruz Biotech, Santa Cruz, CA) were used to direct chromatin antibody pull-down at 4°C overnight (2 μg of antisera per sample). Control goat IgG was prepared from normal goat serum using a Protein A- column. Cross-linking was reversed at 65°C overnight. DNA for PCR analysis was eluted in 50 μl of sterile water. Primers were designed to amplify chromatin DNA spanning P2-promoter GC-box "a" and "b" using MacVector^® ^software (Accelrys, San Diego, CA). GC-box F (5'-TTGAGTGCCAGAGTATGCCCAG), GC-box R (5'-CACCCCAAATGGAGCAAGTG). PCR: 94°C for 3 min (94°C for 20 sec; 56°C for 30 sec; 72°C for 30 sec) and repeated for 36 cycles; and finally terminated at 4°C. PCR products were electrophoresed through a 2% agarose gel and visualized with ethidium bromide staining.

### Cell Culture

Prior authorization was obtained through the Institutional Animal Care and Use Committee- IACUC (UCSF) for all experiments and protocols requiring the use of rat tissues. Primary cultures of rat neonatal DRG neurons were isolated and maintained in media containing NGF (100 ng/ml) as previously described [14]. PC12 cells from American Type Culture Collection (ATCC, Manassas, VA) were maintained in F-12 K (Kaighn's Modification, Gibco-Invitrogen Corp., Rockville, MD) supplemented with 10% heat inactivated horse serum, 5% heat inactivated fetal bovine serum (FBS), streptomycin (100 μg/ml), and penicillin (100 units/ml). HEK293 cells (ATCC) were grown in Dulbecco's modified Eagle's medium (DMEM) H-21 supplemented with 10% heat inactivated FBS, streptomycin (100 μg/ml) and penicillin (100 units/ml) (Cell Culture Facility, UCSF).

### Dual Luciferase Reporter Assay

Neonatal DRG neurons and PC12 cells were plated onto coated 96-well plates (Nunc, Naperville, IL) as previously described [14]. In either case, each sample was composed of 50 μL of Opti-MEM^® ^I (Cell Culture Facility, UCSF) combined with 0.70 ng of Lipofectamine™ 2000 (Invitrogen, Carlsbad, CA) and allowed to incubate for 5 minutes at room temperature. 50 μL of Opti-MEM^® ^I was also combined with a total of 0.3 μg/well of the desired pGL3-reporter construct and/or appropriate expression construct. Additionally, the reference *renilla *luciferase reporter plasmid, pRL-SV40 was included at 0.05 μg/well. The Lipofectamine™ 2000 and DNA solutions were then combined following the manufacturer's recommendations (Invitrogen, Carlsbad, CA). Overall, transfection efficiency was < 5% in primary neonatal DRG neurons [14]. When indicated, PC12 cells were cultured in the presence of NGF (100 ng/ml) after transfection. Following 48 hours of culture, cell lysates were prepared according to the manufacturer's recommendations of the Dual Luciferase Reporter Assay System^® ^(Promega, Madison, WI). Both firefly and renilla luciferase products were measured in a MicroLumatPlus^® ^LB96V microplate luminometer using Winglow^® ^software (Perkin-Elmer Berthold, Wellesley, MA). Firefly luciferase activity was normalized to renilla luciferase activity as a relative ratio resulting in a "Relative Luciferase Activity", which represents the transcriptional activity directed by a particular luciferase reporter construct. In experiments where multiple expression plasmids were required, empty control plasmid was used to maintain an equivalent DNA concentration between transfected samples.

### Plasmid Constructs and siRNA

Luciferase reporter plasmids pGL3-E (empty) and pGL3-0.4 kb containing TRPV1 P2-promoter were previously described [14]. Co-expression of Sp1-like transcription factors was accomplished through the transfection of pN3-Sp1, pN3-Sp3, pN3-Sp4 and pN3-Empty, a gift from Prof. G. Suske (Marburg, Germany) [15]. siRNA knockdown experiments were performed through the transfection of the pBS/U6 plasmid based constructs containing targeted short hairpin loops: siRNA scrambled (scr) (gggaattaatatgcacacaggcc) siRNA-Sp1 (gggaacatcaccttgctacct) nucleotides 881-901, accession no. NM_138473 (XM 028606.7) and siRNA Sp4-1: (gggctccaactttaacacctt) nucleotides 1551-1571 accession no. NM_003112. Total plasmid concentrations remained constant between experimental groups through the addition of empty control plasmids. (siRNAs were a gift from G. Gill, Boston, MA) [16].

### Site-directed mutagenesis

**Figure 1:**
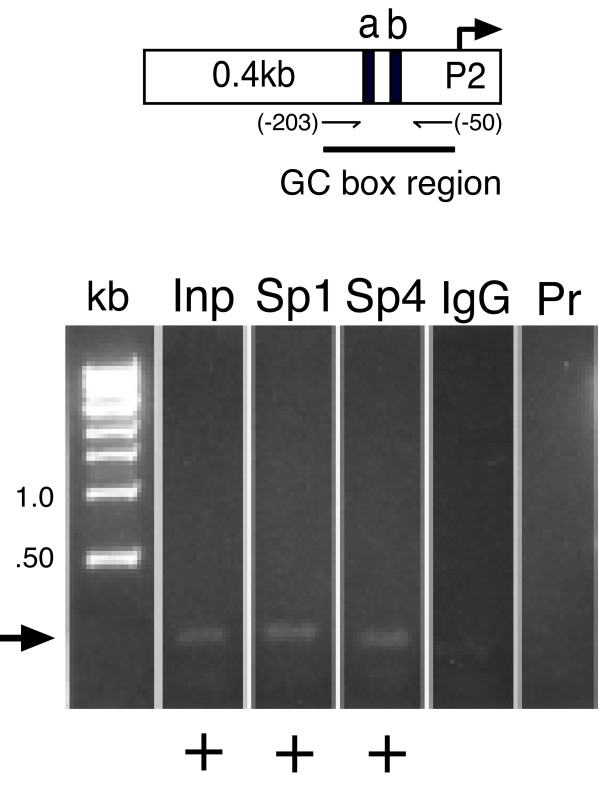
**Transcription factors Sp1 and Sp4 are bound *in vivo *to the GC-box region of P2-promoter in DRG chromatin**. Composite ethidium bromide-stained agarose gel showing evidence of PCR amplified products directed by oligodeoxynucleotide primers spanning TRPV1 GC-box "a" and "b" (top) using template DNA provided as: control input chromatin DNA without immunoprecipitation (Inp), chromatin immunoprecipitated with antisera against transcription factors (Sp1) or (Sp4), chromatin immunoprecipitated with non-immune (IgG), absence of chromatin template - primers only (Pr). Plus sign (+) denotes successful amplification of expected PCR product (arrow). There was no evidence *in vivo *of Sp3 binding (not shown). Each lane represents 1 of at least 3 independent ChIP assays. Molecular size ladder: kilobase (kb).

To delete GC-box "a" and "b" (Figure 1A) residing within the P2-promoter, we utilized the pGL3-0.4 kb luciferase reporter plasmid as template and followed the manufacturer's primer design software (Stratagene, La Jolla, CA) [14]. Using primers: Del-a F (5'-CATCCCTGCCGTACGCCACGAGGACC CTCA); Del-a R (5'-TCTGTGAGGGTCCTCGTGGCGTACGGCAGGGATG); Del-b F (GAGGACCCTCAC AGAGGCACCGGCCACTC); Del-b R (GAGTGGCCGGT GCCTCTGTGAGGGTCCTC), deletion was performed according to the method described in the QuikChange^® ^Site-Directed Mutagenesis Kit (Stratagene, La Jolla, CA). All PCR was performed using pfu Turbo (Stratagene, La Jolla, CA) by initially denaturing the template at 95°C for 30 sec, followed by denaturing at 95°C for 30 seconds, annealing at 60°C for 1 minute, extension at 68°C for 7 minutes, with this cycle repeated 19 times. Original template DNA was digested by Dpn I treatment at 37°C for 1 hour. After 1% agarose gel analysis, desired bands were excised and isolated using the PureLink Quick Gel Extraction Kit (Invitrogen, Carlsbad, CA). Isolated DNA was then ligated using T4 DNA ligase (New England Biolabs, Beverly, MA) and transformed into XL1-Blue 'super-competent' cells (Stratagene, La Jolla, CA). The resultant constructs containing GC-box deletions "a" and "b" were subjected to DNA sequence analysis and only the targeted GC-box binding sites "a" (GGGGAGGGG) and "b" (GGGAGG) were confirmed to be disrupted within the modified 0.4 kb reporter plasmid (Biomolecular Resource Center DNA Sequencing Facility, UCSF, San Francisco, CA).

### Quantitative RT-PCR

#### Electroporation

To obtain a sufficiently high level of transfection efficiency to detect gene expression changes in cultured DRG neurons, we used the Amaxa Nucleofector II Device with the Rat Neuron Nucleofector Kit (Lonza, Basel, CH). For each nucleofection sample, we harvested 40 neonatal DRGs (~1.3 × 10^6 ^cells according to the hemacytometer count). The manufacturer's protocol (Optimized Protocol for Rat Dorsal Ganglion Neurons - Amaxa^®^, Lonza Basel CH) was followed: Samples were transfected with 3 ug of the cDNA expression plasmids: PN3-empty, PN3-Sp1, or PN3-Sp4 [15]; or the knockdown plasmids: siRNA-scramble, siRNA-Sp1, or siRNA-Sp4 #1 [16]. Each sample was transfected using program G-013. Following electroporation, total DRG cultures were plated with a total volume of 2 mL in 24-well plates on 15mm coverslips pre-coated with poly-D-ornithine/laminin (0.1 mg/ml/5 ug/ml). After a three-hour incubation period, the top 1 mL media was replaced with equal volume of fresh media plus 100 ng/ml NGF. Transfection by electroporation with 3 ug Monster™ GFP (Promega, Madison, WI) showed a transfection efficiency of approximately 30-40% in neonatal DRG neurons (data not shown).

#### qPCR

Following RNA purification (TrIzol^®^, Invitrogen, Carlsbad, CA), precipitation and first strand cDNA synthesis (First Strand^®^, Stratagene, San Diego, CA), the level of expression Sp1 and Sp4 mRNAs in rat DRG neurons in culture were analyzed using quantitative real-time PCR performed on the StepOnePlus Real-Time PCR system (Applied Biosystems, Carlsbad, CA). Two experimental approaches were used to manipulate gene expression in the DRGs: siRNA-directed knockdown of Sp1 or Sp4 and over-expression of Sp1 or Sp4 through transfection with Sp1 or Sp4 cDNA. All PCR reactions were performed using 10 μl of TaqMan^® ^Fast Universal Master Mix 2x (Applied Biosystems, Carlsbad, CA), 2 μl 50 ng/μl cDNA, 1 μl of forward and reverse primers, and water to reach the final volume of 20 μl/rxn. PCR was carried out using inventoried primers specific for rat G6PDH from Applied Biosystems (ABI), Carlsbad, CA: (Cat# Rn00566576_ml), Sp1 (Cat# Rn00561953_ml) and Sp4 (Cat# Rn00562717_ml), human Sp1 (Cat# Hs00916521_ml) and Sp4 (Cat# Hs00162095_ml), and custom designed primers for rat TRPV1 Forward: CAA GGC ACT TGC TCC ATT TG; Reverse: TCT GTG GCC CAA TTT CGA; Probe: CCT GCA CCT AGC TGG. Each sample was run as a single-plex reaction system along with a negative control (template: water) for each primer being tested, all samples were run in triplicate. The mRNA expression levels of the genes analyzed were represented as Relative Quantities (RQ) using the comparative C_T _method (RQ = 2^-ΔΔCt^). First, C_T _(threshold cycle) values for each sample and target gene were obtained from real-time PCR analysis with the StepOne^® ^Software (Applied Biosystems, Carlsbad, CA). C_T _values of each gene were then normalized with respect to the housekeeping gene (G6PDH), using the equation where ΔΔC_T _= (C_T, Target _- C_T, G6PDH_) _Sample _- (C_T, Target _- C_T, G6PDH_) [17]. The reference C_T _values were derived from the control (empty vector/scrambled) samples. RQ values of all other treated samples with the same target gene are compared to the control reference values.

### Statistics

Relative luciferase activity was expressed as the mean of three independent experiments each done in at least triplicate measures, +/- SEM. Mean values between groups were compared using ANOVA with Bonferroni post-hoc test (Prism 5.0, GraphPad). P values less than 0.05 were considered to show a significant difference. Differences in mRNA expression levels between non-treated control DRGs and Sp1 or Sp4 over-expession and siRNA knockdowns respectively, were analyzed by two-tailed unpaired t-test with the GraphPad Prism software (GraphPad Software, La Jolla, CA).

## Results

### Sp1 and Sp4 are bound *in vivo *to the GC-box region of TRPV1 P2-promoter

Search for TRPV1 genomic control elements capable of responding to inflammatory mediators revealed no classical response elements within the P2- promoter region [14]. A search for alternative regulatory sites revealed tandem GC-box sites 5' to the P2 transcriptional start site (Figure 1, top). We have termed these two GC-box regions as: GC-box 'a' (GGGGAGGGGC) and GC-box 'b' (GGGAGGCCGGCC) (GenBank: DQ015702). Since Sp1-like transcription factors are known to bind to GC-box sites and activate promoter regions in an NGF-dependent manner [18-21], we first determined if any of the most well studied Sp1-like transcription factors could be expressed in rat DRG by performing a RT-PCR survey of mRNA for factors Sp1-4. mRNA encoding transcription factors Sp1, Sp3 and Sp4 but not Sp2 were identified in rat DRG (data not shown). To determine which of these Sp1-like transcription factors were expressed as protein in DRG and subsequently bound to the endogenous TRPV1 promoter region spanning GC-box "a" and "b", we utilized chromatin immunoprecipitation-ChIP analysis (Methods). ChIP provides for the *in vivo *detection of candidate transcription factors bound to a known promoter region within its native chromatin structure [22-24]. Although we were unable to develop primer sets that individually amplified GC-box "a" versus "b" due to the inherent GC-content and secondary structures (data not shown), it is understood that ChIP analysis provides a superior method (when compared to electrophoretic mobility shift assays - EMSA) to distinguish transcription factor binding that occurs only in the context of the native chromatin structure. This is especially critical given that Sp1-like factors (Sp1, Sp3, Sp4) are reported to have identical binding affinities to isolated GC-box binding targets when studied by EMSA *in vitro *[25].

When sheared chromatin derived from intact DRGs harvested from rats 1.5 months of age or enriched cultures of neonatal DRG neurons were analyzed by ChIP (Methods), we successfully amplified DRG chromatin fragments using antisera directed against Sp1 or Sp4 (Figure 1). Overall, strong evidence for Sp1 (3/4 ChIPs) and Sp4 (3/3 ChIPs) binding were observed (not all gels shown). In contrast, evidence for Sp3 binding was much less convincing (1/3 ChIPs) with a faint band representing the lowest levels of binding detectable amongst the three transcription factors tested (data not shown). When non-specific antiserum (IgG) was used for immunoprecipitation or when PCR amplification was performed without template DNA (primers alone - Pr), either a very faint band of smaller size was observed or no detectable fragment was visualized. Nevertheless, taken together ChIP analysis of rat DRG demonstrates transcription factors Sp1 and Sp4 binding to a region spanning GC boxes "a" and "b" within the P2-promoter. We then sought to understand what functional consequence Sp1-like factors have on TRPV1 promoter activity.

### GC-box "a" is essential for activation of the TRPV1 P2-promoter

**Figure 2:**
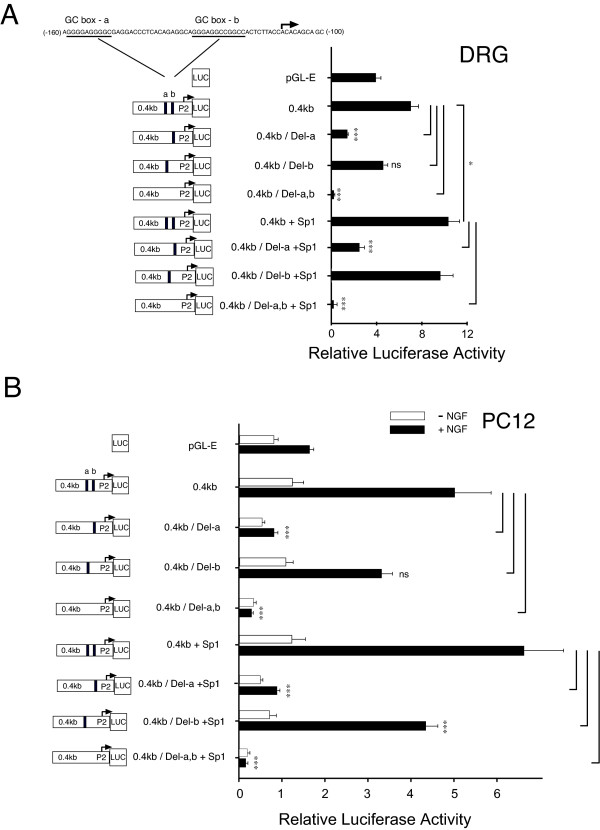
**GC-box "a" is essential for TRPV1 P2-promoter activation in DRG neurons and NGF-treated PC12 cells**. Comparison of P2-promoter activity in DRG neurons + NGF **(A) **or +/- NGF-treated PC12 cells **(B) **directed by: empty pGL3 reporter plasmid (pGL-E); control reporter plasmid (0.4 kb); 0.4 kb reporter with deletion of GC-box "a" (Del-a); 0.4 kb reporter with deletion of GC-box "b" (Del-b) or deletion of both GC-box "a & b". Deletion of GC-box "a" resulted in a complete loss of promoter activity when compared with the (0.4 kb) P2-promoter control in DRG neurons and NGF treated PC12 cells. Deletion of GC-box "b" directed a trend to decrease promoter activity in DRG neurons and NGF treated PC12 cells. Concurrent loss of GC-box a & b resulted in the lowest measurable promoter activity. When identical experiments were repeated under conditions of human Sp1 cDNA (A,B) over-expression, P2-promoter activity continued to be lost following deletion of GC-box "a" or GC-box "a & b". Loss of GC-box "b" under conditions of Sp1 (A,B) over-expression showed a small decrease of P2-promoter activity that attained significance in NGF treated PC12 cells. Diagram (left) indicates location of GC-box deletions and start site of transcription for P2-promoter expressing firefly luciferase (Luc). Error bars SEM (n = 3) quadruplicate measures. Significant differences: ANOVA (***) p <0.001; (*) p < 0.05.

To establish a functional link between candidate GC-box sites and P2-promoter activation, we examined the effect of their individual deletion on P2-promoter activation in cultured DRG neurons and on NGF-dependent promoter activity in cultured PC12 cells. As shown in Figure 2A, when the luciferase reporter construct 0.4 kb containing P2-promoter was transfected into cultured DRG neurons and luciferase activity was measured 48 hours later (Methods), a robust increase in transcriptional activity was observed when compared with the empty vector control. Following the selective deletion of GC-box "a", P2-associated promoter activity directed by the 0.4 kb reporter was completely lost. When GC-box "b" was deleted but GC-box "a" remained intact, there was a small decrease that did not reach significance. As shown in Figure 2B, when identical experiments were conducted in an established model of NGF action - PC12 cells, we observed the previously reported NGF-dependent increase in P2-promoter activity following NGF treatment [14]. However, we also observed the loss of NGF-dependent promoter activity with the deletion of GC-box "a" and a small decrease in NGF dependent activity with deletion of GC-box b that did not reach significance. When both GC-box "a+b" were deleted, the lowest observed level of promoter activity was obtained. These experiments suggest that GC-box "a", is essential for P2-promoter activation in DRG neurons as well as NGF-dependent transcription in PC12 cells. In contrast, GC-box "b", may have a modulatory role in DRG neurons given that its loss is associated with a trend to diminish promoter activity in DRG neurons.

**Figure 3:**
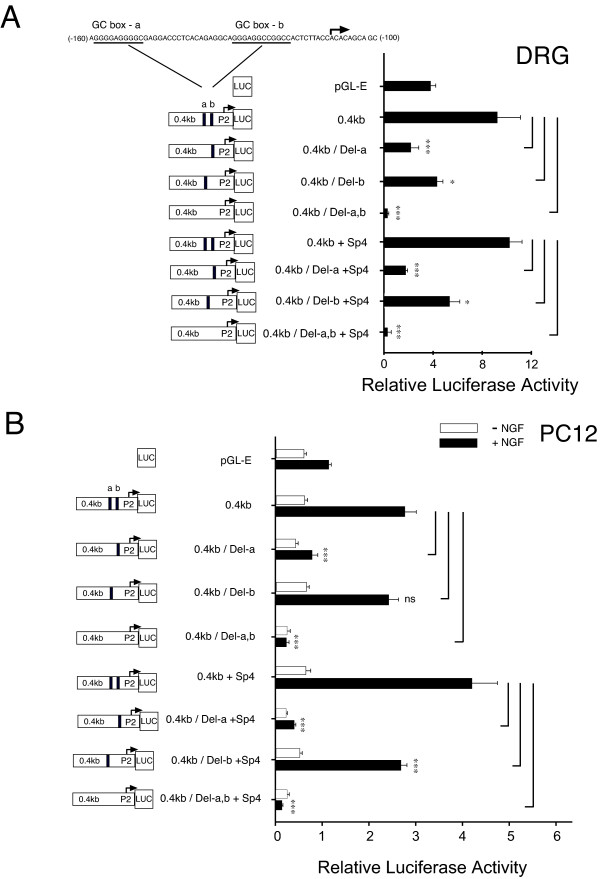
**GC-box "b" modulates TRPV1 P2-promoter activity**. Comparison of P2-promoter activity in DRG neurons + NGF **(A) **or +/- NGF-treated PC12 cells **(B) **directed by: empty pGL3 reporter plasmid (pGL-E); control reporter plasmid (0.4 kb); 0.4 kb reporter with deletion of GC-box "a" (Del-a); 0.4 kb reporter with deletion of GC-box "b" (Del-b) or deletion of both GC-box "a & b". When experiments were repeated under conditions of human Sp4 cDNA over-expression (A,B), P2-promoter activity continued to be lost following deletion of GC-box "a" or GC-box a & b. Loss of GC-box "b" under conditions of Sp4 (A,B) over-expression showed a small decrease of P2-promoter activity that was most evident in NGF treated PC12 cells. Diagram (left) indicates location of GC-box deletions and start site of transcription for P2-promoter expressing firefly luciferase (Luc). Error bars SEM (n = 3) quadruplicate measures. Significant differences: ANOVA (***) p < 0.001; (*) p < 0.05.

The complete loss of promoter activity with deletion of GC-boxes "a+b" suggests that within the P2-promoter, no additional (cryptic) regulatory sites capable of promoter activation exist beyond GC-box "a & b". Given the evidence that transcription factor Sp1 is bound to the P2-promoter (Figure 1), we then repeated this series of experiments under conditions of Sp1 over-expression. As shown in Figure 2A, co-transfection of Sp1-cDNA with the P2-promoter construct 0.4 kb directed an increase in promoter activity. However, deletion of GC-box "a", or GC-box "a + b", again resulted in a complete loss of promoter activity whereas deletion of GC-box "b" did not show significant change in promoter activity. Similar results were observed in parallel experiments conducted in NGF treated PC12 cells (Figure 2B). An identical series of experiments was completed, now including conditions of Sp4 over-expression instead of Sp1 over-expression in cultured DRG neurons and NGF treated PC12 cells (Figure 3A,B). Although there was a trend for increased promoter activity under conditions of Sp4 over-expression in NGF treated PC12 cells, it did not reach significance. As previously observed under conditions of Sp1 over-expression, loss of promoter activity following deletion of GC-box "a" or GC-box "a + b" was not reversed by Sp4. Interestingly, deletion of GC-box "b" in this series was now associated with a statistically significant decrease in promoter activity in cultured DRG neurons and NGF treated PC12 cells.

### Sp1 and Sp3 increase TRPV1 P2-promoter activity in cultured DRG neurons

**Figure 4:**
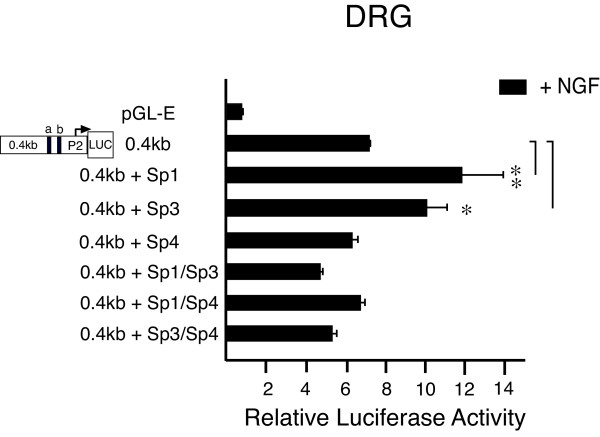
**Transcription factors Sp1 and Sp3 increase P2-promoter activity in DRG neurons**. P2**-**promoter reporter plasmid (0.4 kb) directs an ~8 fold increase in luciferase activity when compared with the empty reporter control (pGL-E). When construct 0.4 kb is co-transfected with a plasmid expressing Sp1, or Sp3, a significant increase in promoter activity was observed. However, co-transfection of Sp4 or a combination of Sp1/Sp3, Sp1/Sp4 or Sp3/Sp4 (equal ratios) failed to increase promoter activity beyond what was observed with the 0.4 kb alone. Error bars SEM (n = 3) triplicate measures. Significant differences: ANOVA (**) p < 0.01, (*) p < 0.05.

Given evidence of Sp1, Sp4 and possibly small amounts of Sp3 - binding to the GC-box region, we then sought to determine what effect the over-expression of these Sp1-like factors would have on P2-promoter (0.4 kb) - directed promoter activity in cultured DRG neurons. As shown in Figure 4, we again observed the expected increase in promoter activity following transfection of the 0.4 kb construct [14]. Co-transfection of expression plasmids encoding Sp1 or Sp3 expression plasmids with the 0.4 kb construct directed a further increase in promoter activity. On the other hand, co-transfection of the Sp4 expression plasmid did not show a significant increase. Interestingly, when Sp1 was paired with Sp3 or Sp4, no increase in promoter activity was observed, as was also observed when Sp3 was paired with Sp4. These results suggest that transcription factor Sp1 positively regulates TRPV1 P2-promoter activity and the presence of other members of the Sp1-like family (Sp3, Sp4) may serve to modulate or compete for control of transcription at the TRPV1 gene P2-promoter.

### An inhibitor of Sp1-like transcription factors dose-dependently blocks NGF and Sp1- dependent TRPV1 promoter activity in PC12 cells

**Figure 5:**
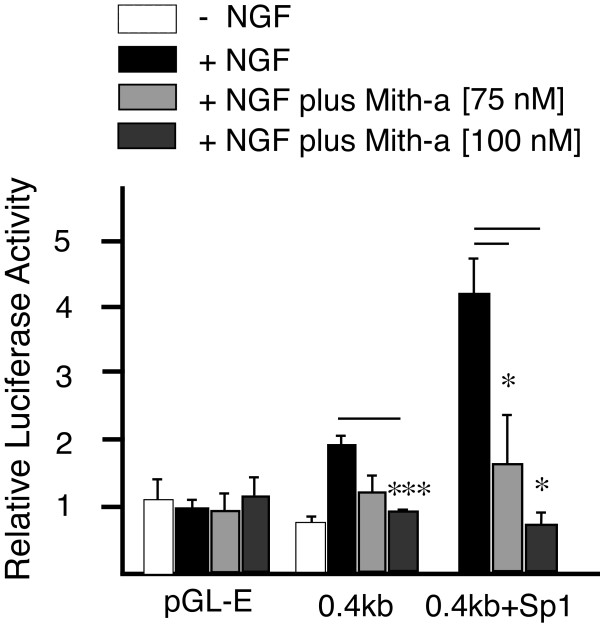
**An inhibitor of Sp1 (mithramycin-a) dose-dependently blocks NGF and Sp1- dependent P2-promoter activity in PC12 cells**. P2-promoter reporter plasmid (0.4 kb) directs an ~2 fold increase in luciferase activity when treated with NGF × 48 hours [14]. Treatment with an inhibitor (mithramycin-a) of Sp1 function that disrupts GC-box/transcription factor binding, blocked the NGF-dependent P2-promoter activity. When the experiment was repeated in the presence of co-transfected Sp1, the expected increase in activity directed by Sp1 was dose-dependently inhibited by mithramycin-a. Error bars SEM (n = 3) triplicate measures. Significant differences: ANOVA (***) p < 0.001, (*) p < 0.05.

To further establish the role of Sp1-like transcription factors in the regulation of TRPV1 promoter activation, we then asked whether a known inhibitor of Sp1 function could disrupt P2-promoter activity in a model of NGF-dependent TRPV1 transcription. As previously observed [14], NGF increased P2-promoter activity in PC12 cells (Figure 5, 0.4 kb black bars); however, mithramycin-a, an inhibitor of Sp1 function [26-29] dose-dependently blocked the NGF-induced promoter activity. Importantly, mithramycin-a also dose-dependently blocked Sp1-dependent increases in P2-promoter activity (Figure 5). Similar results were observed for Sp3 (data not shown). Attempts to perform identical experiments in neonatal DRG neurons were unsuccessful due to mithramycin-a associated toxicity and the requirement of NGF to sustain viability of neonatal DRG neurons, not seen with PC12 cells. Although the inhibitory effect of mithramycin-a does not preclude disruption of other Sp1-like member binding to GC-box binding sites, it does support the idea that in part NGF- dependent transcription at P2-promoter is mediated by Sp1 and/or other Sp1-like transcription factors.

### siRNA directed knockdown of Sp1 decreases P2-promoter activity in DRG neurons and NGF-dependent activity in PC12 cells

**Figure 6:**
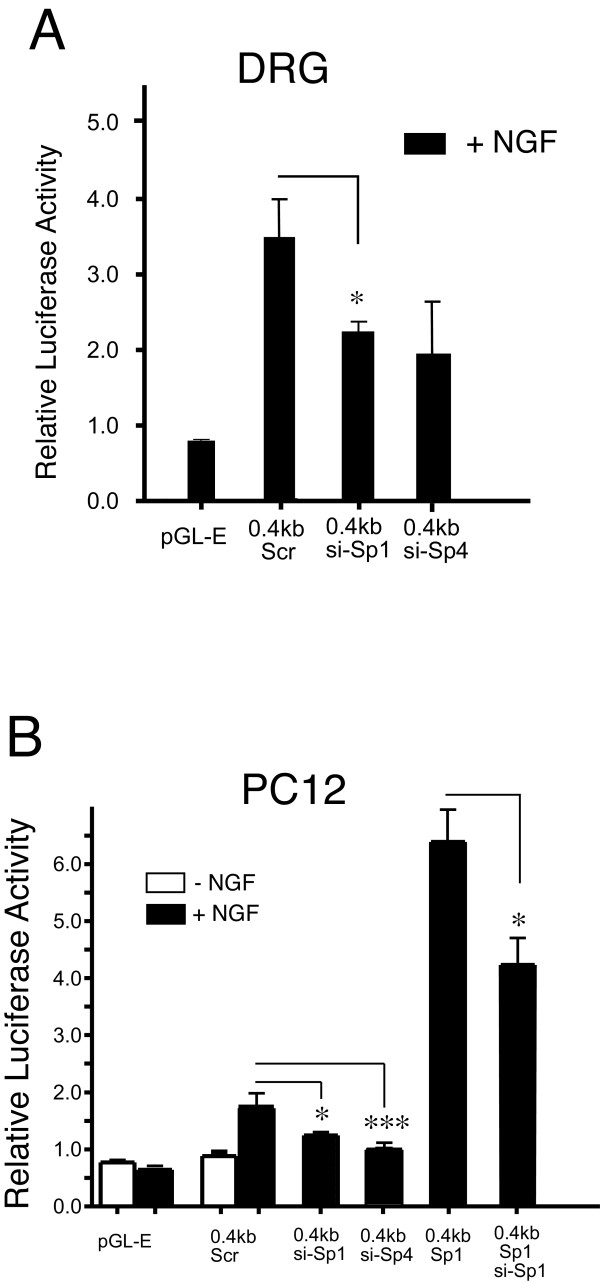
**P2-promoter activity in DRG neurons is decreased by Sp1-siRNA and Sp1 and Sp4 siRNA also block P2 promoter activity in NGF treated PC12 cells**. Co-transfection of Sp1-siRNA with the 0.4 kb P2-promoter construct resulted in a significant decrease in promoter activity when compared with co-transfection of the scrambled (scr) siRNA control whereas Sp4-siRNA co-transfection failed to show a decrease (A). In contrast, both Sp1-siRNA and Sp4-siRNA co-transfection experiments showed a significant decrease in NGF treated PC12 cells (B). Primary cultures of NGF-treated dorsal root ganglion (DRG) neurons were transfected with either (pGL-E) empty luciferase reporter plasmid; **(**0.4 kb + pBS) Luciferase reporter containing the P2-promoter plus empty siRNA vector pBS/U6; (0.4 kb + siRNA-Sp1**) **0.4 kb plus siRNA construct containing the Sp1 directed hairpin encoding Sp1 nucleotides 881-901; (0.4 kb + siRNA-Sp4) 0.4 kb plus siRNA construct containing the Sp4 directed hairpin encoding Sp4 nucleotides 1551-1571. (siRNAs were a gift from G. Gill Lab, Tufts, Boston) [16] . The presence of the scrambled siRNA control plasmid reduced the expected promoter activity of the 0.4 kb reporter plasmid in DRG. Co-transfection of the Sp1 cDNA in PC12 cells (0.4 kb + Sp1) directed a further increase in P2-promoter activity that was significantly reversed by co-transfection of the Sp1-siRNA construct. Error bars SEM (n = 3) triplicate measures. Significant differences: ANOVA (*) p < 0.05; (***) p < 0.001.

Finally, we sought to demonstrate the dependence of endogenous Sp1 and Sp4 transcription factors on the activation of the P2-promoter through the use of a siRNA knockdown strategy previously shown to decrease Sp1 and Sp4 in primary cerebellar granule neurons [16]. Search for off-site hits matched only the Sp1 and Sp4 sequence in a BLAST search of the NCBI nucleotide database (not shown). Although the low transfection efficiency using lipofectamine (≤ 5%) in DRG neurons precluded quantitative analysis of Sp1 or Sp4 content following siRNA treatment, the utility and fidelity of these probes have been previously reported [16] and we have subsequently validated the efficacy of DNA constructs for over-expression or siRNA knockdown at the mRNA level following electroporation (see below). As shown in Figure 6A, co-transfection of siRNA-Sp1 (Gift from G. Gill) into cultured DRG neurons significantly reduced P2-promoter activity directed by the 0.4 kb reporter plasmid. Co-transfection of siRNA-Sp4 showed a trend to decreased levels of promoter activity that did not reach significance. Similar findings were observed when promoter activity was studied in transfected PC12 cells. As shown in Figure 6B, NGF again directed an expected increase in 0.4 kb reporter activity whereas co-transfection of Sp1-siRNA decreased the NGF-dependent promoter activity. Moreover, under conditions of Sp1 over-expression (Figure 6B, 0.4 kb Sp1), the additional increase in promoter activity directed by Sp1 was significantly reversed by co-transfection of Sp1-siRNA. In addition, co-transfection of siRNA-Sp4 also produced a decrease in promoter activity in NGF-treated PC12 cells.

### Over-expression of Sp1 or Sp4 increase endogenous levels of TRPV1 mRNA in cultured DRG neurons

**Figure 7:**
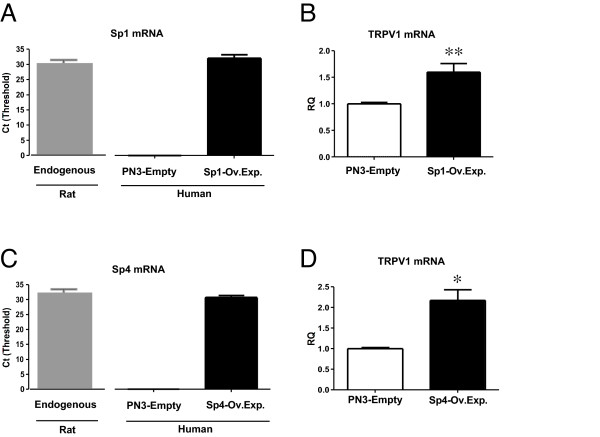
**Over-expression of transcription factor Sp1 and Sp4 mRNA increase endogenous TRPV1 mRNA in cultured DRG neurons**. **(A) **Measurement of endogenous levels of rat Sp1 mRNA in cultured DRG neurons following transfection with empty/PN3 vector (left). Additional expression of an equivalent amount of human form of Sp1 mRNA was achieved following transfection with hSp1/PN3. **(B) **Endogenous TRPV1 mRNA levels were increased following over-expression of hSp1 in cultured DRG neurons (**) p < 0.005. **(C) **Measurement of control levels of rat Sp4 mRNA in cultured DRG neurons following transfection with empty/PN3 vector (left). Additional expression of an equivalent amount of human form of Sp4 mRNA was also achieved following transfection with hSp4/PN3. **(D) **Endogenous TRPV1 mRNA levels increased following over-expression of hSp4 in cultured DRG neurons (*). Error bars SEM (n = 3) triplicate measures. Two tailed unpaired t-test. Significance: (p < 0.05). Ct threshold values were derived from quantitative RT-PCR amplification of cultured rat DRG neuron RNA - see Methods.

Building on our observations that Sp1 and Sp4 are bound to the TRPV1 P2-promoter region *in vivo *and regulate P2-promoter activity, we then attempted to manipulate Sp1 or Sp4 expression in cultured DRG neurons to determine their subsequent downstream effects on changes in endogenous TRPV1 mRNA expression. Given that our lipofectamine-based transfection of DRG neurons and PC12 cells provide relatively small ( < 5%) transfection efficiencies (Methods), we elected to conduct these experiments in cultured DRG neurons following electroporation (Methods) to provide greater transfection rates (30-40%) based on GFP staining in viable cells at 24-48 hours (not shown). As shown in Figure 7A, we first quantified endogenous levels of rat Sp1 mRNA in cultured DRG neurons following transfection with the empty expression plasmid PN3. Because no amplification of the Sp1/Sp4 genes for the reference control sample occurs and 2^--ΔΔCt ^analysis cannot be utilized, C_T _values are used instead of RQ values to compare mRNA expression levels (Methods). We then measured the resultant mRNA content of human Sp1 mRNA following either electroporation with the empty PN3 vector versus a human Sp1/PN3 expression plasmid (previously used in promoter activity assays) and compared its C_T _value with the C_T _value associated with baseline levels of the rat Sp1 or Sp4 mRNA.

Human Sp1-like transcription factors and their cognate cDNAs differ slightly in nucleotide sequence, but encode indistinguishable functional properties across species. Therefore, we were able to independently measure and compare the additional contribution of human Sp1 mRNA. As shown in Figure 7A, following transfection with the human Sp1 cDNA, an approximately equal amount of human Sp1 mRNA in addition to the endogenous rat Sp1 mRNA was detected in cultured DRG neurons. Therefore, following an approximate doubling of Sp1 mRNA, we observed a significant increase in endogenous TRPV1 mRNA (Figure 7B). In like manner, we repeated these experiments but measured the endogenous expression of rat Sp4 mRNA (Figure 7C) and subsequently human Sp4 mRNA content in cultured DRG neurons following electroporation of the Sp4 cDNA. Again, we observed an approximate doubling of Sp4 mRNA with a corresponding significant increase in TRPV1 mRNA (Figure 7D).

### Double knockdown of transcription factors Sp1 and Sp4 directs a decrease in TRPV1 mRNA in cultured DRG neurons

**Figure 8:**
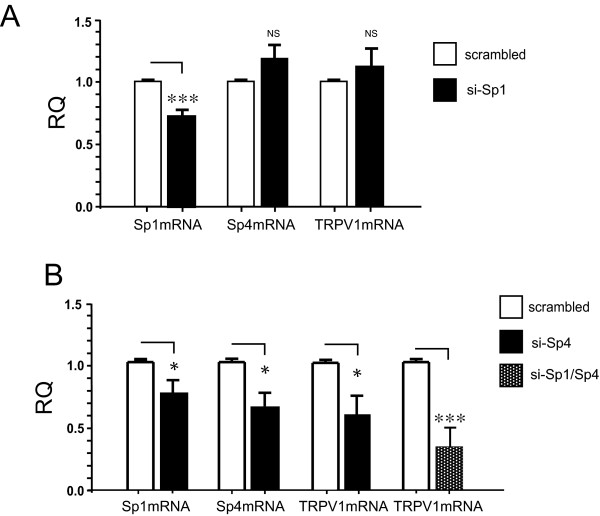
**Knock-down of transcription factors Sp1/Sp4 decrease endogenous TRPV1 mRNA in cultured DRG neurons**. **(A) **Evidence of Sp1mRNA knockdown following transfection of siSp1 in cultured DRG neurons (***) p < 0.0001 (left). Apparent changes in Sp4 (middle) or TRPV1 mRNA (right) were not significant (ns). **(B) **Transfection of siSp4 resulted in an apparent knockdown of Sp1, Sp4 and TRPV1 mRNA. Combined knockdown of Sp1 + Sp4 using an equal ratio (1:1) of siSp1/siSp4 resulted in the most consistent knockdown of endogenous TRPV1 mRNA. Error bars SEM (n = 3) triplicates measures. Two tailed unpaired t-test. Significant differences: (*) p < 0.05; (***) p < 0.0001. RQ values of siRNA treated DRGs are compared relative to the RQ values of scrambled controls which represent baseline amounts of Sp1, Sp4 or TRPV1 mRNA following transfection of a scrambled siRNA or Spx control vector, see Methods for details.

Having observed the generally positive regulatory effects of Sp1 and Sp4 on TRPV1 mRNA expression in cultured DRG neurons (Figure 7), we returned to a siRNA knockdown strategy to help confirm the relationship between Sp1, Sp4 and TRPV1 RNA transcriptional control. As shown in Figure 8A, following electroporation of cultured DRG neurons with Sp1-siRNA, a significant decrease in Sp1 mRNA was detected when compared with control experiments conducted with a scrambled Sp1-like siRNA control vector. However, no significant changes were observed in concurrently measured Sp4 mRNA or TRPV1 mRNA content. When parallel experiments with siSp4 - mediated knockdown were conducted, a decrease in Sp4 mRNA was observed. Importantly, when Sp4 mRNA knockdown was achieved, there was evidence of a concurrent decrease in Sp1 and TRPV1 mRNA (Figure 8B). Finally, given the apparent "cross-talk" between Sp1 and Sp4 gene expression, we electroporated an equal ratio (1:1) of siSp1 plus siSp4 and observed a significant decrease in TRPV1 mRNA in cultured DRG neurons (Figure 8B).

## Discussion

The regulation of transcription is a fundamental way that mammalian cells restrict and adjust their gene expression in response to changing cellular conditions such as stress or injury. In the case of primary afferent nociceptors, gene expression of ion channel -receptors such TRPV1 can be increased or decreased depending on changes in the cellular environment. Recruitment of RNA polymerase II for gene-specific transcription of TRPV1 DNA into RNA is envisioned to require a unique set of transcription factors that direct a subset of sensory neurons to express TRPV1. Our findings reported here support the hypothesis that the P2-promoter is a major site for the regulation of TRPV1 RNA transcription. In addition to being adjacent to TRPV1 transcriptional (RNA) start sites confirmed by 5'-RACE and EST database [14], we have also reported that the P2-promoter directs transcription in a cell-type specific manner. Moreover, P2-promoter activity is increased in DRG cultures enriched in sensory neurons but lacking in cells such as 3T3 fibroblasts [14] or HEK293 cells (unpublished observations). We now report that factors Sp1/Sp4, acting at a specific GC-box binding site, play a critical role in controlling TRPV1 RNA transcription in sensory neurons.

### Transcription factors Sp1 and Sp4 regulate TRPV1 P2-promoter activity

Two functionally distinct GC-box binding sites have been identified within the P2-promoter. We propose that Sp1 and Sp4 function to regulate activation of TRPV1 transcription primarily through binding to GC-box "a" in the P2-promoter. Evidence to support this hypothesis includes: Detection of Sp1 and Sp4 protein bound to native DRG chromatin structure of the TRPV1 P2-promoter region GC-box a + b (Figure 1); Complete loss of promoter activity in DRG neurons and NGF-treated PC12 cells following deletion of GC-box "a" alone or both GC-box "a + b" (Figure 2,3); Increased promoter activity in DRG neurons co-transfected with Sp1 (Figure 2A,4); Blockade of NGF/Sp1- dependent promoter activity in PC12 cells using an inhibitor of Sp1-like binding, mithramycin-a (Figure 5); Decreased promoter activity in DRG neurons/NGF treated PC12 cells with siSp1 and decrease of Sp1-dependent promoter activity with siSp1 and siSp4 in PC12 cells (Figure 6).

### Factors Sp1/Sp4 regulate TRPV1 RNA transcription in sensory neurons

Building on our observations of Sp1/Sp4 - dependent changes in promoter activity, we then determined whether manipulation of Sp1/Sp4 mRNA expression would direct concomitant changes in endogenous levels of TRPV1 mRNA in sensory neurons. As shown in Figure 7, we demonstrated that over-expression of Sp1/Sp4 mRNA in cultured DRG neurons was associated with an increase in TRPV1 mRNA. Conversely, we demonstrated a decrease in TRPV1 mRNA in sensory neurons under conditions of siSp4 and siSp1/Sp4 RNA knockdown (Figure 8B). Importantly, in both experimental series, changes in Sp4 were associated with the largest change in TRPV1 mRNA content. This suggests that although both Sp1 and Sp4 are bound to the P2-promoter site and are purported to have similar DNA binding properties *in vitro*, Sp4 may provide the dominant contribution to activate TRPV1 transcription in sensory neurons.

### The structure and function of Sp1-like transcription factors

Sp1, one of the first transcription factors to be isolated [30], is the founding member of an expanding family of Sp1-like/Kruppel-like transcription factors that share common structural features such as glutamine-rich N-terminal activation domains and C-terminal zinc-finger DNA binding domains [31-33]. Members of this Sp1-like family are distinguished by their ability to bind GC- and / or GT-rich DNA regions within promoter regions and to activate gene transcription. Sp1 is also one of the best-characterized transcription factors [31,32]. Although Sp1 was initially considered to be 'ubiquitously' expressed, its level of expression actually differs greatly (up to a 100 fold) during development and between tissue/cell types [32,34]. A multitude of expressed genes common to all cells have been proposed to be regulated by Sp1 by virtue of containing GC/GT box binding sites within or adjacent to their promoter region. However, the actual number of genes critically dependent upon Sp1 are much fewer, suggesting sophisticated roles in its maintenance of differentiated cell types and tissue function. The role of Sp1 in nociception is unstudied as null mice completely lacking Sp1 die early in embryogenesis [35], whereas, Sp3 -/- null mice are growth-retarded and die at birth due to respiratory failure [18,36]. Nevertheless, in addition to TRPV1, other Sp1-regulated genes may be involved in nociception such as the NR1 promoter [18,36], DRG-specific expression of H-Antigen [37] and expression of 12 (S) lipoxygenase - products that have been shown to mediate bradykinin induced TRPV1 activation [5,38].

Sp1-like factors share a high degree of homology, but they do not appear to have merely redundant functional attributes. Despite the ability of Sp1, 3 and 4 to bind identical 'GC-box' consensus targets *in vitro*, a more selective pattern of Sp1-like binding and transcriptional activation is actually observed when studied in the context of a complex genomic sequence, complex chromatin structure or diverse cellular environments. This is consistent with our observations that neither Sp3 nor Sp4 appear to exhibit the same profile of expression/activation as found for Sp1. Under control conditions, we observed only trace amounts of endogenous Sp3 protein bound to DRG TRPV1 P2-promoter. However, over-expression of Sp3 resulted in an increase in TRPV1 promoter activity (Figure 4). In contrast, co-expression of Sp3 with Sp1 resulted in a reduction of Sp1-mediated TRPV1 promoter activation. Therefore, as opposed to a synergistic effect reported between the tandem binding of Sp1, increased levels of Sp3 may serve a negative regulatory role in TRPV1 transcription. Such a role is consistent with a previous report showing that Sp3 inhibits the transcriptional activation of Sp1 [39].

### Does transcription factor Sp4 play a unique role in regulating nociceptor phenotype?

Sp4 (HF-1b) is distinguished from Sp1 and Sp3 by a restrictive pattern of expression in neuronal cell types [39,40]. Depending on the cellular context, there is also evidence that Sp4 can function as a transcriptional activator but without the capacity to act in a synergistic manner as exhibited by Sp1. Sp4 has also been reported to be expressed in other sensory-neuronal systems such as the cGMP - phosphodiesterase beta subunit that is exclusively expressed in rod photoreceptors [41]. There are no published reports focused on the consequence of reduced Sp4 expression on behaviors related to peripheral nociception. Although Sp4 is highly expressed in several subregions of the brain, Sp4 -/- null mice showed only selective structural defects in the hippocampus [42]. Behavioral testing of mice with reduced levels of Sp4 (2-5% of residual activity) showed structural defects in the hippocampus as well as contextual memory deficits and sensori - motor gating abnormalities [43]. Mice modified with a deletion of the Sp4 N-terminal activation domain appear normal at birth but the majority later died by 1 month of age [44,45]. Importantly, Sp4 -/- null mice appear to be predisposed to cardiac arrhythmias leading to sudden death [46]. When Sp4 was reduced to 60% of expressed levels in neural crest derived cells (primary sensory, sympathetic and parasympathetic neurons), physiologic defects in atrial and atrial-ventricular conduction were found, despite a lack of morphological changes in cardiac tissues [47]. This may reflect the loss of specific ion channels and/or other elements that are essential to signal transduction including synaptic structure and dendritic remodeling [16]. Although it remains to be studied what affect the Sp4 null phenotype will have on the regulation of peripheral pain transduction, our findings here suggest Sp4 playing a critical role in the gene expression of TRPV1 mRNA in DRG neurons.

### Recruitment of Sp1-like transcription factors is dependent on post-translational modification

In addition to a diverse pattern of tissue expression, Sp1 and Sp4 are subjected to a complex array of post-translational modifications that regulate target site (DNA) binding and/or recruitment of other transcription factors at gene promoter sites. These modifications include glycosylation, phosphorylation, acetylation and sumolyation [16,48-50]. Post-translational modification of transcription factors is a primary way by which extracellular signaling events have long-term consequences on target gene expression. For example, Sp1 was found to be a critical downstream regulator of NR1 promoter activity in response to NGF activation of the extracellular signal regulated kinase -1 (ERK-1) cascade and the action of phosphitidylinositol 3-kinase (PI3 K) [19]. It is interesting to consider that NGF-induced activation of PI3 K has been shown to not only translocate PI3 K to the nucleus for transcriptional activation, but also to direct NGF-induced sensitization of TRPV1 thermal hyperalgesia through receptor modification and increased trafficking of TRPV1 to the plasma membrane [51-53]. It is plausible that such NGF dependent pathways could drive both acute (receptor sensitization) and chronic (increased transcription/translation) changes in nociceptor phenotype.

NGF has emerged as a key inflammatory mediator directing both acute and persistent pain and hyperalgesia [12,13]. As previously published by us and others, elevated concentrations of NGF drive an increase in TRPV1 mRNA expression in cultured DRG neurons and an increased number of TRPV1 mRNA positive DRG neurons *in vivo *[54-57]. This is consistent with our earlier findings that NGF stimulates P2-promoter activity [14]. In this present study, NGF-dependent P2-promoter activity requiring the presence of an intact GC-box "a" (Figure 2D) is blocked by a known inhibitor of Sp1 function (Figure 4) and can be decreased by Sp1-siRNA (Figure 5B). Taken together, these findings support the hypothesis that Sp1, in part, mediates NGF-dependent TRPV1 transcription. In the central nervous system, Sp1-like transcription factors direct protective regulatory responses under cellular stress and injury [58]. Sp1 has also been shown to direct the expression of NGF dependent cell survival genes [20]. In fact, Sp1 and Sp3 are oxidative stress-induced transcription factors in cortical neurons that function to reduce apoptosis and positively regulate neuronal survival [59]. However, it remains to be studied what role Sp1 and its related family of transcription factors play under conditions of peripheral inflammation and nerve injury.

## Conclusions

**Figure 9:**
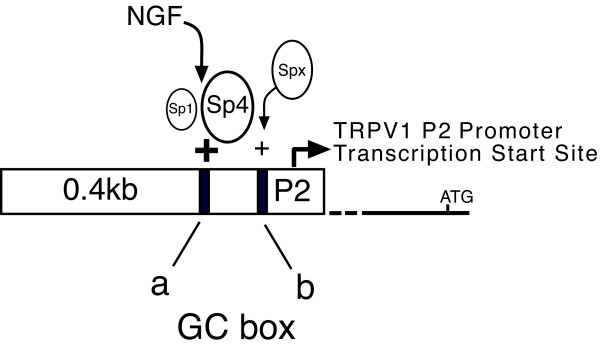
**Regulation of TRPV1 transcription at the rat P2-promoter**. TRPV1 P2-promoter contains two tandem GC-box binding sites adjacent to the start site of TRPV1 transcription (arrow). GC-box "a" was found to be essential for transcriptional activation and appears to be the primary regulatory site in the P2-promoter and is co-regulated by factors Sp1 and Sp4. Depending on the cellular environment and potential state of transcription factor abundance/modification, this model proposes factor Sp4 playing a dominant role in the activation of TRPV1 transcription amongst the Sp1-like factors examined in this study. One type of transcription factor activation may arise from the activity of exogenous products of inflammation, such as NGF. Sp1, Sp4 and/or other members of the Sp1-like family (Spx) may also bind to GC-box region "b" providing additional modulation and full transcriptional activation. Classically transcriptional regulation is dynamic and rapidly responds to intrinsic and extrinsic changes of the cellular milieu. It is envisioned that transcriptional control is directed by a combination of protein modifications and/or formation of a multi-protein transcription factor complex to attract and activate RNA polymerase II (not shown). Differing 'sizes' of transcription factors represent their relative contribution to activation of TRPV1 transcription.

We propose an initial model of rat TRPV1 gene expression that is dependent on factors Sp1/Sp4, with Sp4 playing a critical role in activating TRPV1 transcription (Figure 9). This idea is persuasive because the non-neuronal cell line HEK293 directs no TRPV1 P2-promoter activity unless Sp4 is co-transfected (unpublished observations). Sp4 may also form a complex with Sp1 and/or other members of the Sp1-like family. Recently, another factor, Sp5, has been reported and was found to repress Sp1 target genes such as p21 [60]. The idea that Sp5 may also participate in TRPV1 transcription is attractive because Sp5 shows a restricted pattern of expression involving the trigeminal ganglion and dorsal portion of the spinal cord, although its role in DRG remains unreported [61]. Ultimately, investigating the role of Sp4 and other Sp1-like transcription factors using transgenic models will help reveal their contribution to the initiation and/or maintenance of painful hyperalgesic states observed under conditions of tissue injury.

## Competing interests

The authors declare that they have no competing interests.

## Authors' contributions

CC participated in the development and performance of the ChIP and luciferase-based transcriptional assays, performed statistical analysis and helped compose draft portions of the manuscript. KZ participated in the development and performance of the luciferase-based transcriptional assays, over-expression and siRNA knockdown experiments as well as performed statistical analysis and helped compose draft portions of the manuscript; AF participated in the development, performance and statistical analysis of the quantitative RT-PCR measurements. JL participated in the development, performance and statistical analysis of the quantitative RT-PCR measurements. HE participated in the development, performance and statistical analysis of the quantitative RT-PCR measurements; QX participated in the early development of the study related to the role of Sp1, constructed luciferase reporter plasmids used throughout the study; MS conceived and directed this study, coordinated the experimental design, conducted and reviewed the statistical analysis and drafted the manuscript. All authors read, revised and approved the final manuscript.
